# Conditional disruption of *Nr5a1* directed by *Sox9-Cre* impairs adrenal development

**DOI:** 10.1038/s41598-024-63264-9

**Published:** 2024-05-29

**Authors:** Ayako Tagami, Yayoi Ikeda, Kyoko Ishizuka, Mamiko Maekawa

**Affiliations:** Department of Anatomy, School of Dentistry, Aichi Gakuin University, 1-100 Kusumoto-cho, Chikusa-ku, Nagoya, Aichi 464-8650 Japan

**Keywords:** Nr5a1, Conditional knockout, SOX9, Adrenal development, Mouse, Developmental biology, Endocrinology

## Abstract

The current study aimed to investigate the effect of *Sox9-Cre-*directed *Nr5a1*-conditional knockout (*Sox9-Cre;Nr5a1*^*flox/flox*^) on adrenal development. We showed that SOX9 is expressed by adrenocortical cells at E10.5–E11.5 but is extinguished no later than E12.5. The number of adrenocortical cells significantly reduced in *Sox9-Cre;Nr5a1*^*flox/flox*^ mice while the number of cleaved caspase 3-positive cells increased compared to that in the controls at E11.5–E12.5, when the adrenal primordium (AP) is about to expand. This indicated that fetal adrenocortical cells are lost via apoptosis due to *Nr5a1* ablation by E12.5. Both medulla formation and encapsulation were perturbed, accompanied by a smaller AP size, in *Sox9-Cre;Nr5a1*^*flox/flox*^ mice during embryonic development. Adult *Sox9-Cre;Nr5a1*^*flox/flox*^ adrenals were hypoplastic and exhibited irregular organization of the medulla with aberrant sex differentiation in the X zone. Additionally, there were histologically eosin-negative vacuolated cells, which were negative for both the X-zone marker 20αHSD and the steroidogenesis marker 3βHSD at the innermost cortex of *Sox9-Cre;Nr5a1*^*flox/flox*^ adrenals. Although *Nr5a1*^+*/−*^ adrenals were hypoplastic, a small number of chromaffin cells were properly located in the center, having normal sex differences in the X-zone. The results collectively provided in-vivo evidence that *Nr5a1* plays a critical role in AP expansion and subsequent adrenal development.

## Introduction

The adrenal gland is a steroidogenic organ essential for homeostasis in mammals, and its formation is a complex process during embryonic development^1–3^.

In mice, the first event is the establishment of the adrenogonadal primordium (AGP), the common primordium of the adrenal gland and the gonads, via thickening of the coelomic epithelium around embryonic day (E) 9.5. Adrenocortical progenitors differentiate in the AGP and migrate to form the adrenal primordium (AP) positioned dorsomedial to the gonadal primordium (GP) at E10.5–E11. Thereafter, the AP expands and initiates the developmental program of adrenal gland organogenesis at E12.5. Simultaneously, neural crest-derived cells arrive at the AP, start invading the cortex, and differentiate into chromaffin cells. Capsule formation occurs through the condensation of mesenchymal cells surrounding the AP. The fetal cortex is gradually replaced by the adult (definitive) cortex. At birth, the medulla, located as an island in the center, is surrounded by the cortex, which is divided into the zona glomerulosa (ZG), zona fasciculata (ZF), and X-zone in mice. The X-zone develops in both sexes after birth but degenerates at puberty in males and at first pregnancy in females or in old nulliparous females; however, the role of the X-zone and the mechanisms controlling its maintenance or regression still remain unclear.

Nuclear receptor subfamily 5 group A member 1 (Nr5a1, also known as SF1 and Ad4BP), a member of the nuclear receptor superfamily, plays an essential role in the development and function of tissues involved in the endocrine/reproductive system, including the adrenal gland, gonad, anterior pituitary, and ventromedial hypothalamus^4^. *Nr5a1*/NR5A1 expression occurs in the urogenital ridge, where AGP is formed as early as E9.5, and is maintained in both the adrenal gland and the gonad during development and adulthood^5,6^. In *Nr5a1*^−/*−*^ mice, AP and GP disappeared due to apoptosis around E11.5–E12, just before the start of adrenal organogenesis^7^. In *Nr5a1*^+*/−*^ mice, adrenal glands are hypoplastic, although gonadal development is not affected^8^. In contrast, the overexpression of NR5A1 produces adrenocortical hyperplasia and tumors, which express gonadal markers^9^. These studies collectively suggested that *Nr5a1* expression is critical for adrenal and gonadal development and function^10^.

SRY-box containing gene 9 (Sox9) is well known as a key factor in testis differentiation, interacting with SRY, the testis-determining gene on the Y-chromosome, and NR5A1 specifically in the XY GP to induce supporting cells into Sertoli cells^11^. We had previously studied the importance of Nr5a1 in early testis differentiation using a conditional knockout (cKO) of *Nr5a1* in *Sox9-Cre* mice and found that the XY gonads from *Sox9-Cre;Nr5a1*^*flox/flox*^ mice reduced the number of Sertoli cells to less than 10% compared to that in control mice by E12.5, resulting in partial male-to-female gonadal sex reversal (ovotestes) in adulthood^12^. Before upregulation in the XY gonad, *Sox9*/SOX9 is expressed at low levels in somatic (mesenchymal) cells of the genital ridges and other tissue primordia, irrespective of sex, and plays a crucial role in cell proliferation and differentiation^13,14^. If Sox9 is expressed by mesenchymal cells in the AP, adrenal development may be affected by *Sox9-Cre*-directed *Nr5a1* disruption. In this study, we aimed to verify the above possibility and investigate the effect of *Sox9-Cre*-directed *Nr5a1* cKO on adrenal development.

## Results

### SOX9 is expressed by adrenocortical cells at E10.5–E11.5 but not after E12.5

**Figure 1:**
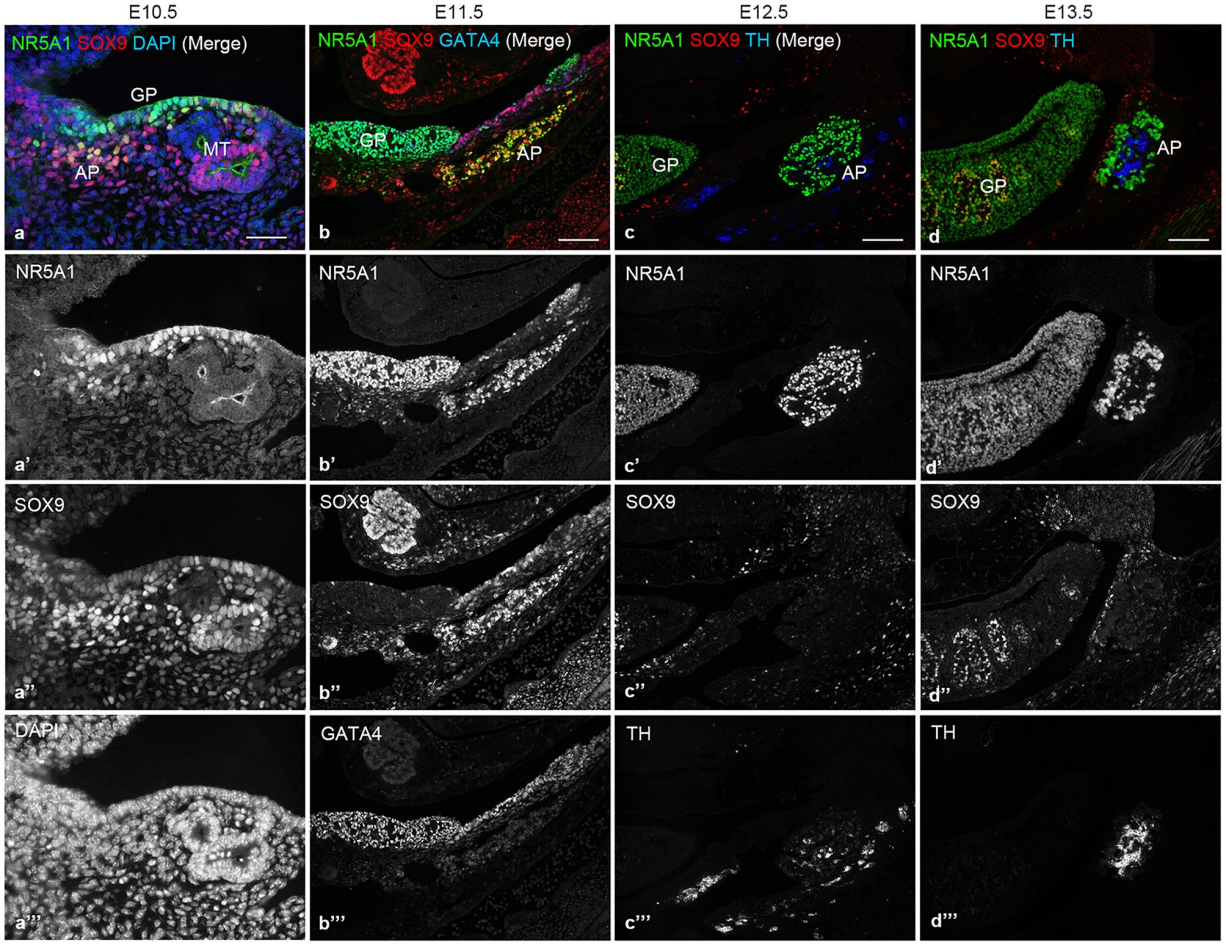
Expression of NR5A1 and SOX9 in the adrenal primordium during early embryonic development. (**a**) Representative images of double-label immunofluorescence for NR5A1 (green) and SOX9 (red) performed on transverse sections of adrenal primordium (AP) from control mice at E10.5. Nuclei are stained blue with DAPI. (**b**–**d**) Representative images of triple-label immunofluorescence for NR5A1 (green), SOX9 (red), and GATA4 (blue) or TH (blue) on parasagittal sections from control mice at E11.5, E12.5, and E13.5. **a**, **b**, **c**, and **d** are merged images of **a**′-**a**′′′, **b**′–**b**′′′, **c**′–**c**′′′, and **d**′–**d**′′′, respectively. GP, gonadal primordium; MT, mesonephric tubules. Scale bars, 50 µm in **a**, 100 µm in **b**–**d**.

At E10.5–E11.5, the AP consists of condensed mesenchymal cells corresponding to adrenocortical cells, which are marked by *Nr5a1*/NR5A1. *Nr5a1*/NR5A1 expression is maintained in both the adrenal and gonadal tissues during the development through adulthood. In contrast, the expression of GATA-binding protein 4 (GATA4), an essential factor for the formation of AGP, is maintained high in gonadal cells while being remarkably downregulated in adrenocortical cells after the formation of AP^8^. We first investigated whether SOX9 is expressed in the early stages of adrenal development using immunofluorescence (IF) with markers for adrenal and gonadal cells. At E10.5, SOX9-positive cells were present in the AP and NR5A1/SOX9 double-positive cells were observed (Fig. 1a). At E11.5, triple-label IF for NR5A1, SOX9, and GATA4 showed that the AP was marked by NR5A1-positive GATA4-negative cells, whereas the GP was marked by NR5A1/GATA4 double-positive cells, and several NR5A1-positive cells co-expressed SOX9 in the AP (Fig. 1b). Around E12.5, the chromaffin precursors arrive at the AP and begin to invade the cortex. Next, we performed triple IF for NR5A1, SOX9, and tyrosine hydroxylase (TH), a marker of chromaffin precursors, on parasagittal sections at E12.5 and E13.5. In the AP, a cluster of NR5A1-positive cells intermingled with some TH-positive cells, but no SOX9-positive cells were detected at either stage (Fig. 1c, d). The results indicated that SOX9 is expressed in the AP at E10.5 and E11.5, but is extinguished no later than E12.5.

## *Sox9-Cre-*directed* Nr5a1* cKO decreases adrenocortical cells and increases apoptosis at early stages of adrenal development

**Figure 2:**
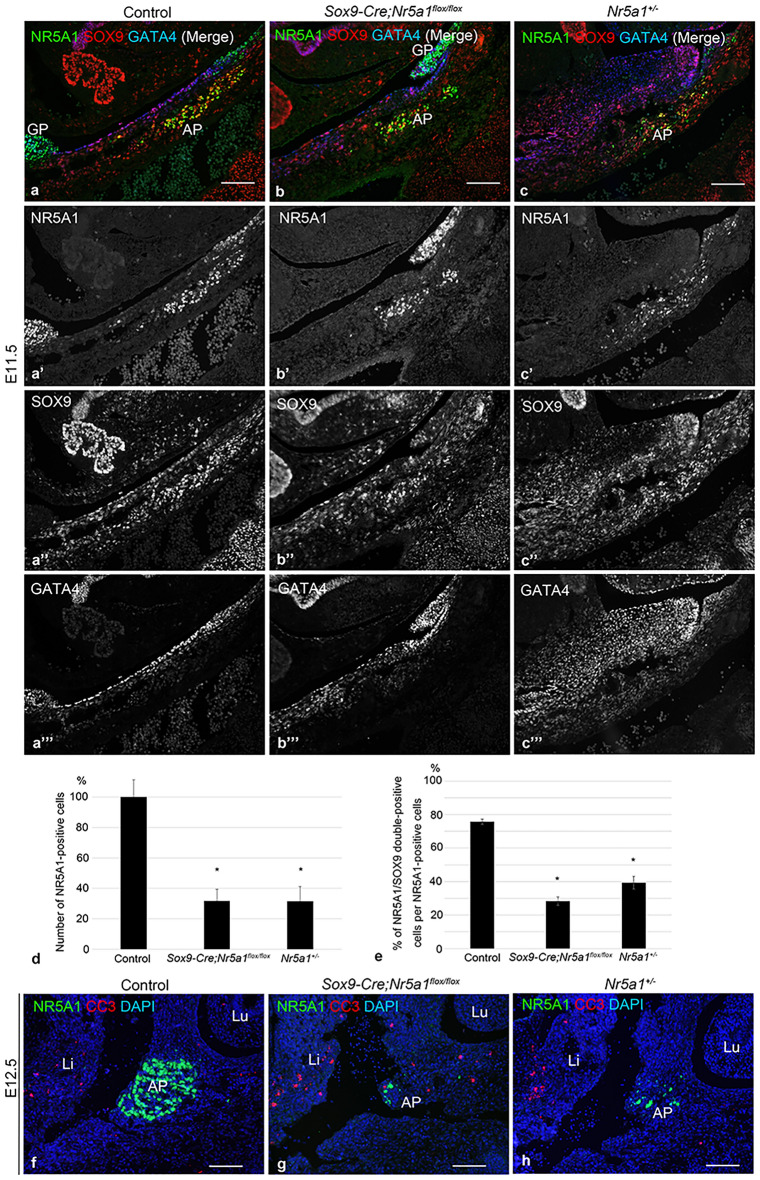
Decreased cortical cells and increased apoptosis in the adrenal primordium during early embryonic development. (**a**–**c**) Representative images of triple-label immunofluorescence for NR5A1 (green), SOX9 (red), and GATA4 (blue) performed on parasagittal sections of embryos at E11.5. **a**, **b**, and **c** are merged images of **a**′–**a**′′′, **b**′–**b**′′′, and **c**′–**c**′′′, respectively. AP, adrenal primordium; GP, gonadal primordium. Scale bars, 100 µm. (**d**) Number of NR5A1-positive adrenocortical cells at E11.5 (Values for control are set at 100%). Data are shown as means ± SEM (n = 3). *P < 0.05 vs. control. (**e**) Proportion (%) of SOX9-positive cells among NR5A1-positive adrenocortical cells at E11.5. Data are shown as means ± SEM (n = 3). *P < 0.05 vs. control. (**f**–**h**) Representative images of double-label immunofluorescence for NR5A1 (green) and cleaved caspase 3 (CC3, red) at E12.5. Nuclei are stained blue with DAPI. In **a**-**c** and **e**–**g**, left panels, middle panels, and right panels are from control, *Sox9-cre;Nr5a1*^*flox/flox*^, and *Nr5a1*^+*/−*^ mice, respectively. AP, adrenal primordium; Li, liver; Lu, lung. Scale bars, 100 µm.

In our previous study, we had detected a remarkable reduction in Sertoli cell progenitors in XY gonads of *Sox9-Cre;Nr5a1*^*flox/flox*^ mice by E12.5^12^. We determined whether *Sox9-Cre*-mediated ablation of *Nr5a1* could simultaneously decrease the number of adrenocortical cells. Since early adrenal development has been reported to be severely impaired in *Nr5a1*^+*/−*^ mice^8^, we examined the *Nr5a1*^+*/−*^ adrenals. Triple IF for NR5A1, SOX9, and GATA4 showed that NR5A1/SOX9 double-positive cells were present in the AP of all three genotypes at E11.5 (Fig. 2a–c). Counting the number of NR5A1-positive cells revealed a loss of 68% of adrenocortical cells in *Sox9-Cre;Nr5a1*^*flox/flox*^ mice compared to that in controls at E11.5 (Fig. 2d), indicating that Cre activity efficiently deleted both *Nr5a1* conditional alleles at the same time. As reported previously^8^, the adrenal size of *Nr5a1*^+*/−*^ mice was smaller than that of controls (Fig. 2c), and the loss of NR5A1-positive cells in *Nr5a1*^+*/−*^ adrenals was 68% of that in the controls at E11.5 (Fig. 2d). The proportion of NR5A1/SOX9 double-positive cells among NR5A1-positive adrenocortical cells at E11.5 were 76%, 28%, and 39% in control, *Sox9-Cre;Nr5a1*^*flox/flox*^, and *Nr5a1*^+*/−*^ mice, respectively, being significantly lower compared to those in control mice while not significantly different from those in *Nr5a1*^+*/−*^ mice (Fig. 2e).

At E12.5, NR5A1-positive cells formed a distinct oval shape of the AP in control mice (Figs. 1c, 2f), whereas only a few NR5A1-positive cells were detected in the corresponding AP area in *Sox9-Cre;Nr5a1*^*flox/flox*^ and *Nr5a1*^+*/−*^ mice (Fig. 2g, h). To determine whether apoptosis contributes to the reduction in adrenocortical cells in *Sox9-Cre;Nr5a1*^*flox/flox*^ mice, we performed double IF for NR5A1 and cleaved caspase 3 (CC3), a marker of apoptotic cells. Few CC3-positive cells were detected in control adrenal glands (Fig. 2f) and in the AP area of *Nr5a1*^+*/−*^ mice (Fig. 2h), whereas abundant CC3-positive cells were observed in the AP area of *Sox9-Cre;Nr5a1*^*flox/flox*^ embryos (Fig. 2g) at E12.5. These results indicated that apoptosis was responsible for the loss of adrenocortical cells in *Sox9-Cre;Nr5a1*^*flox/flox*^ mice.

**Figure 3:**
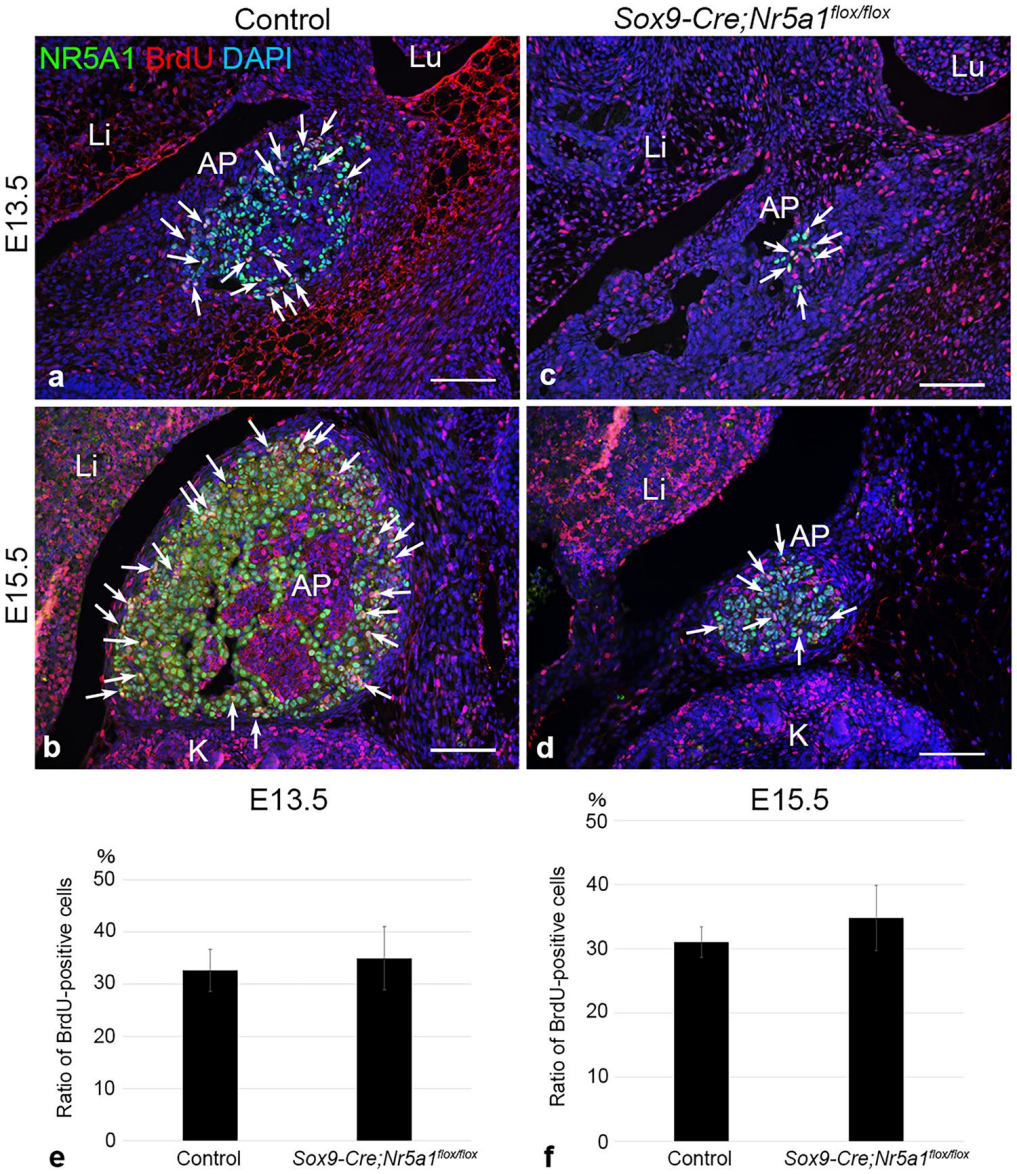
Proliferation of adrenocortical cells during embryonic development. Cell proliferation was examined by BrdU incorporation at E13.5 and E15.5. (**a**–**d**) Representative images of double-label immunofluorescence for NR5A1 (green) and BrdU (red) performed on parasagittal sections of embryos from control (**a**, **b**) and *Sox9-Cre;Nr5a1*^*flox/flox*^ (**c**, **d**) mice at E13.5 and E15.5. Nuclei are stained blue with DAPI. Arrows indicate BrdU/NR5A1 double-positive cells. AP, adrenal primordium; K, kidney; Li, liver; Lu, lung. Scale bars, 100 µm. (**e**, **f**) Percentages of BrdU-positive cells in NR5A1-positive cells in the AP at E13.5 and E15.5. Data are shown as means ± SEM (n = 3). *P < 0.05 vs. control.

To determine whether cell proliferation was affected in *Sox9-Cre*-directed *Nr5a1* cKO, we examined bromodeoxyuridine (BrdU) incorporation by injecting BrdU at 50 mg/kg of body weight into pregnant mice 2 h before euthanasia. Double IF for NR5A1 and BrdU showed that BrdU/NR5A1 double-positive cells were mostly located in the outer area of the AP in control mice at E13.5 and E15.5 (arrows in Fig. 3a, b), whereas they were irregularly distributed in the AP without particular localization in *Sox9-Cre;Nr5a1*^*flox/flox*^ mice (arrows in Fig. 3c, d). The proportion of proliferating adrenocortical cells in *Sox9-Cre;Nr5a1*^*flox/flox*^ mice did not differ from that in control adrenal glands at E13.5 or E15.5 (Fig. 3e, f).

### Adrenal medulla formation and encapsulation are compromised in* Sox9-Cre;Nr5a1*^*flox/flox*^ adrenals

**Figure 4:**
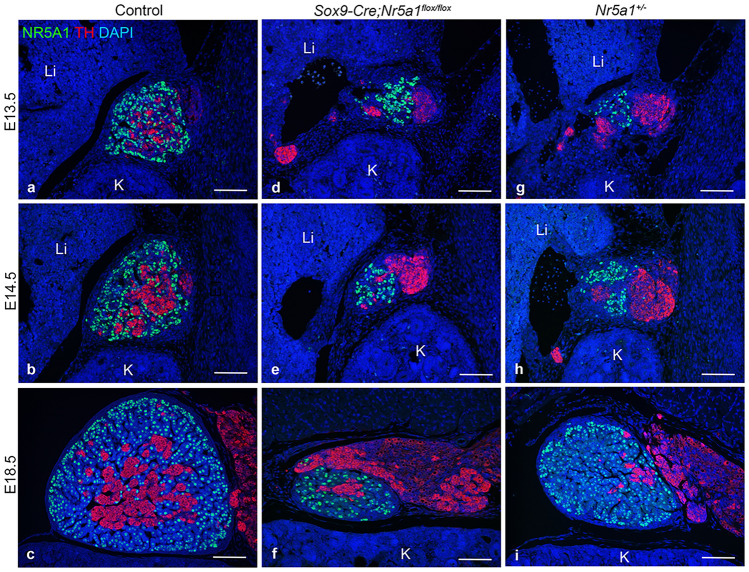
Medulla organization during embryonic adrenal development. Representative images of double-label immunofluorescence for NR5A1 (green) and TH (red) performed on parasagittal sections of embryos from control (**a**-**c**), *Sox9-Cre;Nr5a1*^*flox/flox*^ (**d**–**f**) and *Nr5a1*^+*/−*^ (**g**–**i**) mice at E13.5, E14.5, and E18.5. Nuclei are stained blue with DAPI. K, kidney; Li, liver. Scale bars, 100 µm.

To examine the embryonic adrenal development with medulla formation, double IF for NR5A1 and TH was performed on adrenal sections at stages E13.5–E18.5. Although the number of cortical cells in *Sox9-Cre;Nr5a1*^*flox/flox*^ mice gradually increased from E13.5–E18.5, the adrenal glands in *Sox9-Cre;Nr5a1*^*flox/flox*^ mice were smaller than those in the controls at all stages examined (Fig. 4). The TH-positive cells invading the cortex increased in number and tended to be located in the center of the control adrenal glands from E13.5–E18.5 (Fig. 4a–c), whereas only a small number of TH-positive cells were scattered in the cortex of *Sox9-Cre;Nr5a1*^*flox/flox*^ mice at E13.5–E18.5 (Fig. 4d–f). The adrenal size and dynamics of the TH-positive cells in *Nr5a1*^+*/−*^ mice were similar to those in *Sox9-Cre;Nr5a1*^*flox/flox*^ mice (Fig. 4g–i).

**Figure 5:**
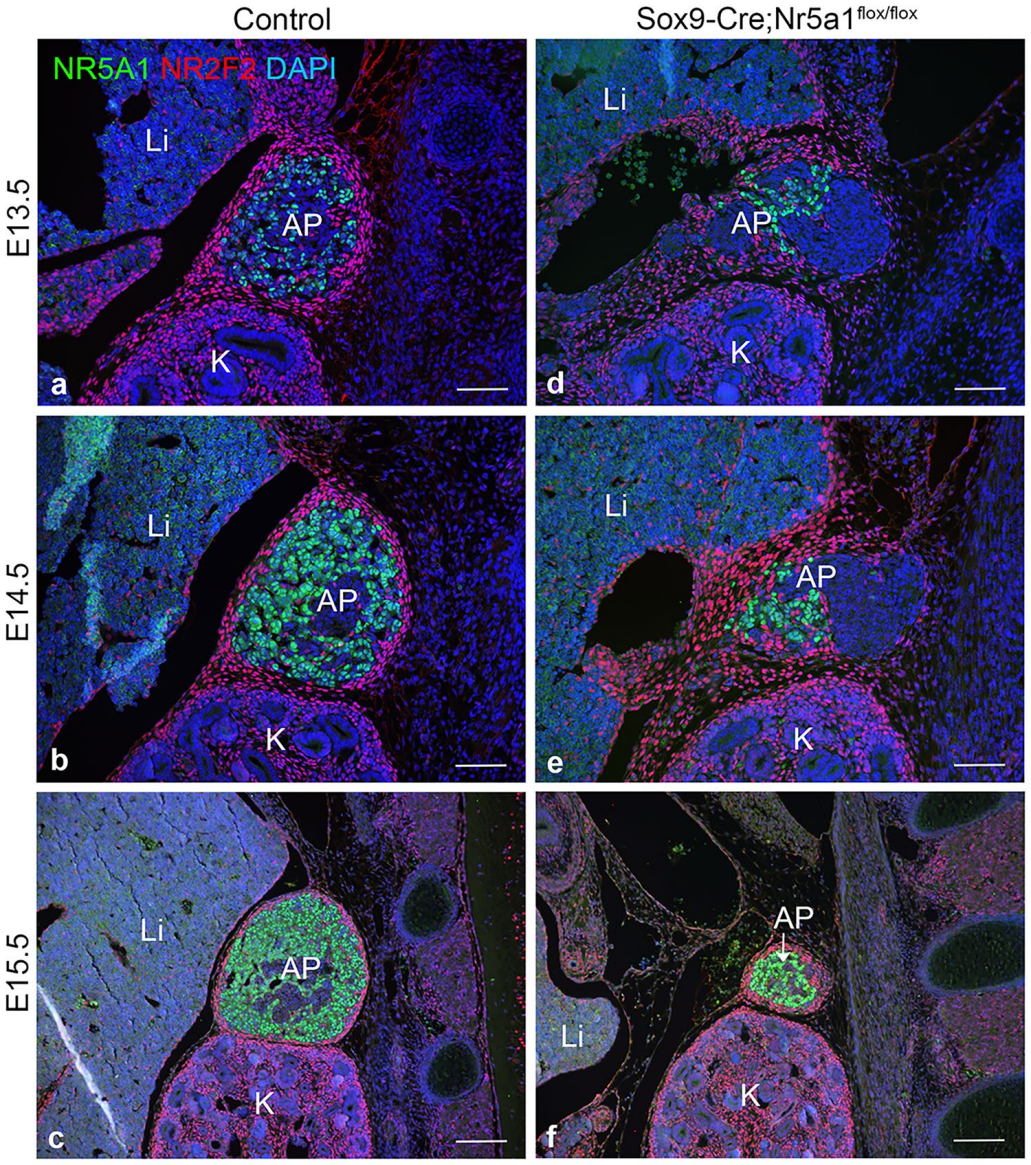
Capsule formation during embryonic development. Representative images of double-label immunofluorescence for NR5A1 (green) and NR2F2 (red) was performed on parasagittal sections of embryos from control (**a**–**c**) and *Sox9-Cre;Nr5a1*^*flox/flox*^ (**d**–**f**) mice at E13.5, E14.5, and E15.5. Nuclei are stained blue with DAPI. AP, adrenal primordium; K, kidney; Li, liver. Scale bars, 100 µm in **a**, **b**, **d**, **e,** 200 µm in **c**, **f**.

To investigate capsular formation, we performed double IF for NR5A1 and nuclear receptor subfamily 2, group f, member 2 (NR2F2, commonly known as CoupTFII), which defines mesenchymal cells, including adrenal capsular cells. NR2F2-positive cells surrounding a mass of NR5A1-positive cells contoured the AP in control mice at E13.5–E15.5 (Fig. 5a–c). In *Sox9-Cre;Nr5a1*^*flox/flox*^ mice, they were irregularly distributed around the AP without forming the typical capsule at E13.5 and E14.5 (Fig. 5d, e). However, at E15.5, a capsular structure was detected in *Sox9-Cre;Nr5a1*^*flox/flox*^ mice (Fig. 5f).

### Postnatal* Sox9-Cre;Nr5a1*^*flox/flox*^ adrenals are hypoplastic with disorganized structures

**Figure 6:**
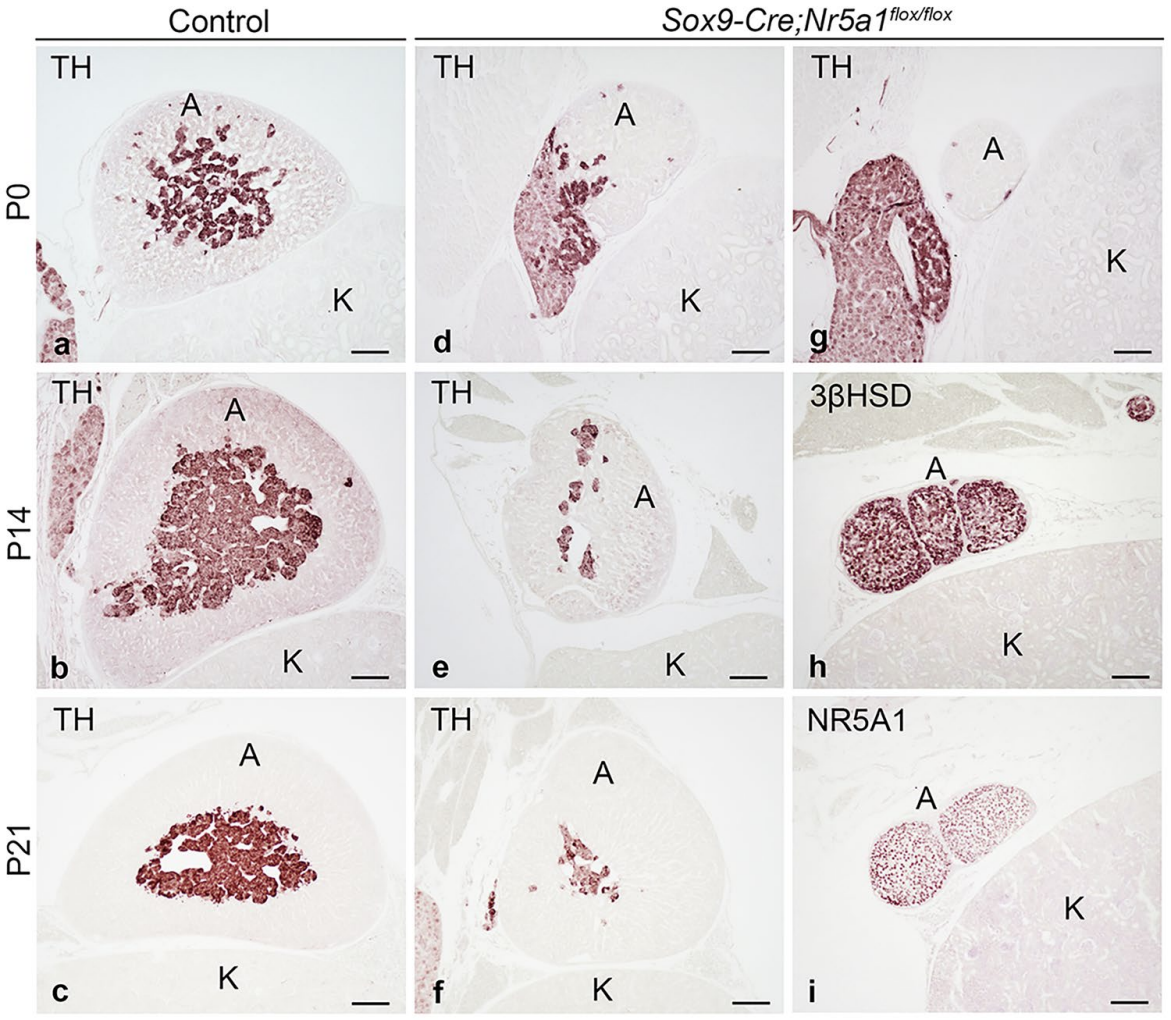
Early postnatal adrenal development. Representative images of immunohistochemistry for TH (**a**–**g**), 3βHSD (**h**), or NR5A1 (**i**) performed on adrenal sections from control (**a**–**c**) and *Sox9-Cre;Nr5a1*^*flox/flox*^ (**d**–**i**) mice at P0, P14, and P21. A, adrenal; K, kidney. Scale bars, 100 µm.

Early postnatal adrenal development was examined at P0, P14, and P21 using immunohistochemistry (IHC). *Sox9-Cre;Nr5a1*^*flox/flox*^ adrenals were smaller than control adrenals at the postnatal stage examined (Fig. 6). TH-positive cells were present as a large mass in the center of control adrenals (Fig. 6a–c), whereas they were lesser in number in *Sox9-Cre;Nr5a1*^*flox/flox*^ mice (Fig. 6d–g). IHC for 3β-hydroxysteroid dehydrogenase (3βHSD), a steroidogenesis marker, or NR5A1 showed that in some cases of *Sox9-Cre;Nr5a1*^*flox/flox*^ mice, the adrenals consisted of cortical cell clusters with few TH-positive cells (Fig. 6h, i).

**Figure 7:**
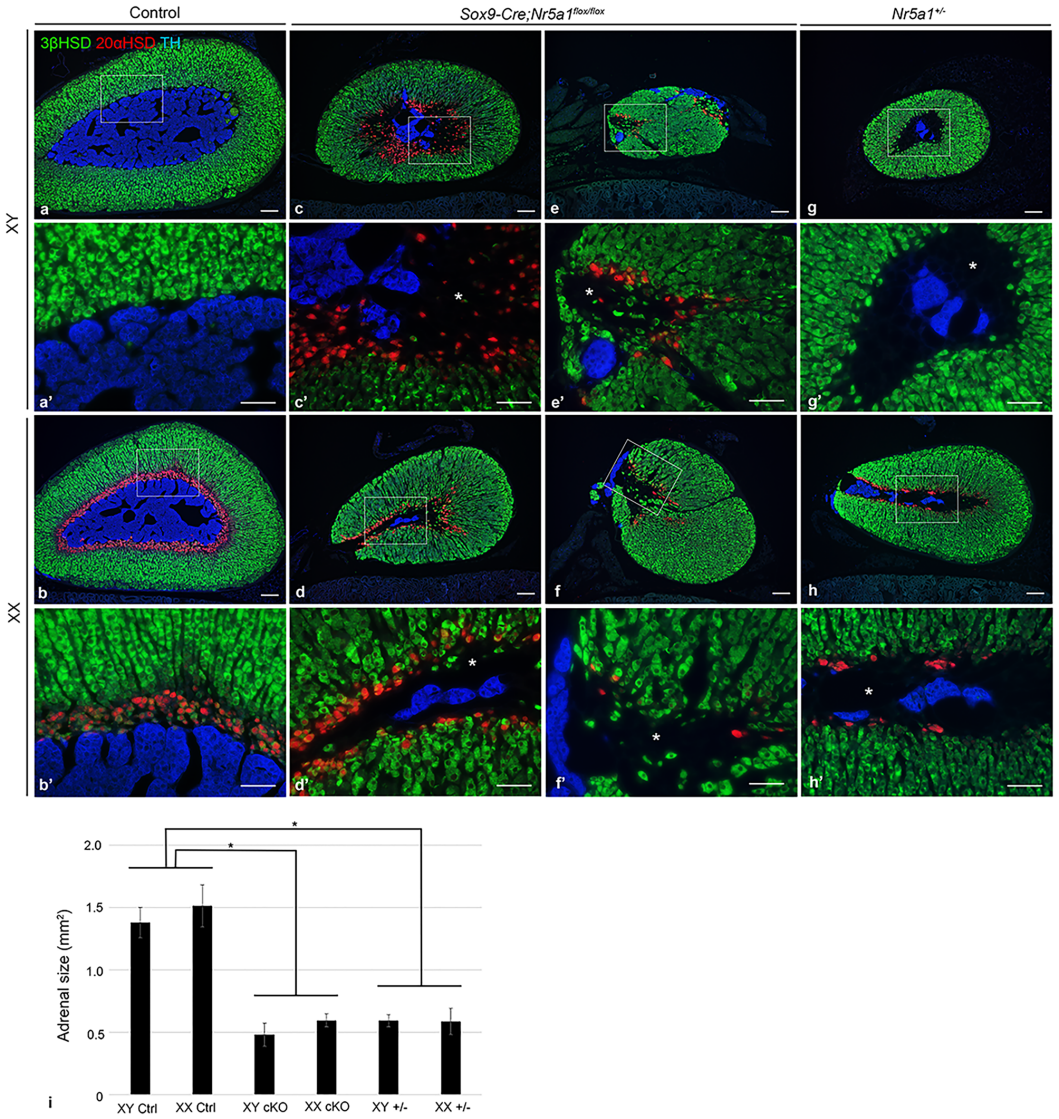
Organization of the cortex, medulla, and X-zone in adult adrenals. Representative images of triple-label immunofluorescence for 3βHSD (green), 20αHSD (red), and TH (blue) performed on adrenal sections from control (**a**, **b**), *Sox9-Cre;Nr5a1*^*flox/flox*^ (**c**–**f**), and *Nr5a1*^+*/−*^ mice (**g**, **h**) of males (XY) and females (XX) at 8 weeks of age. **a**′–**h**′ are higher magnification views of the regions indicated by the box in **a**–**h**, respectively. * indicates the area that is negative for both 20αHSD and 3βHSD, at the innermost cortex in *Sox9-Cre;Nr5a1*^*flox/flox*^ and *Nr5a1*^+*/−*^ adrenals. Scale bars, 100 µm in **a**–**h**, 50 µm in **a**′–**h**′. (**i**) Comparison of adrenal sizes in control, *Sox9-Cre;Nr5a1*^*flox/flox*^, and *Nr5a1*^+*/−*^ mice of XY and XX at 8 weeks of age. Data are shown as means ± SEM (n = 3). *P < 0.05 vs. control.

To learn the effect of *Sox9-Cre*-mediated *Nr5a1* inactivation on adult adrenal organization, triple-label IF staining for 3βHSD, TH, and 20α-hydroxysteroid dehydrogenase (20αHSD), a marker of the X-zone, was performed on the adrenals at 8 weeks of age. A large single mass of TH-positive chromaffin cells was located in the center of the control adrenal glands (Fig. 7a, b). In *Sox9-Cre;Nr5a1*^*flox/flox*^ mice, the adrenals were smaller than those in controls, with the organization of cortex and medulla varying across individual adrenals. TH-positive cells were detected as a smaller mass or several pieces of small islands within the cortex (Fig. 7c–f). In a few *Sox9-Cre;Nr5a1*^*flox/flox*^ adrenals, we failed to detect any portion of the medulla (Supplementary Fig. 1). When adrenal sizes were compared among the three genotypes, *Sox9-Cre;Nr5a1*^*flox/flox*^ mouse adrenals were significantly smaller compared those in controls and were similar to those in *Nr5a1*^+*/−*^ mice (Fig. 7i). Although *Nr5a1*^+*/−*^ adrenals were smaller than the controls, a small island of TH-positive cells was located in the center, surrounded by the cortex, and the adrenal structure was maintained relatively normal (Fig. 7g, h). As reported previously^15^, 20αHSD-positive X-zone cells were detected in females, but not in males, in both the control (Fig. 7a, b) and the *Nr5a1*^+*/−*^ adrenal glands (Fig. 7g, h). In contrast, 20αHSD-positive cells were detected in both sexes of *Sox9-Cre;Nr5a1*^*flox/flox*^ adrenals, although the number of 20αHSD-positive cells varied across individuals (Fig. 7c–f).

Noticeably, we found an area, which was negative for both 20αHSD and 3βHSD, at the innermost side of the cortex in *Sox9-Cre;Nr5a1*^*flox/flox*^ and *Nr5a1*^+*/−*^ adrenals (Fig. 7c′–h′,’ indicated by *) but not in control adrenals (Fig. 7a′, b′). The 20αHSD-positive X-zone cells, which were also detected at the innermost cortex, were faintly positive or negative for 3βHSD.

**Figure 8:**
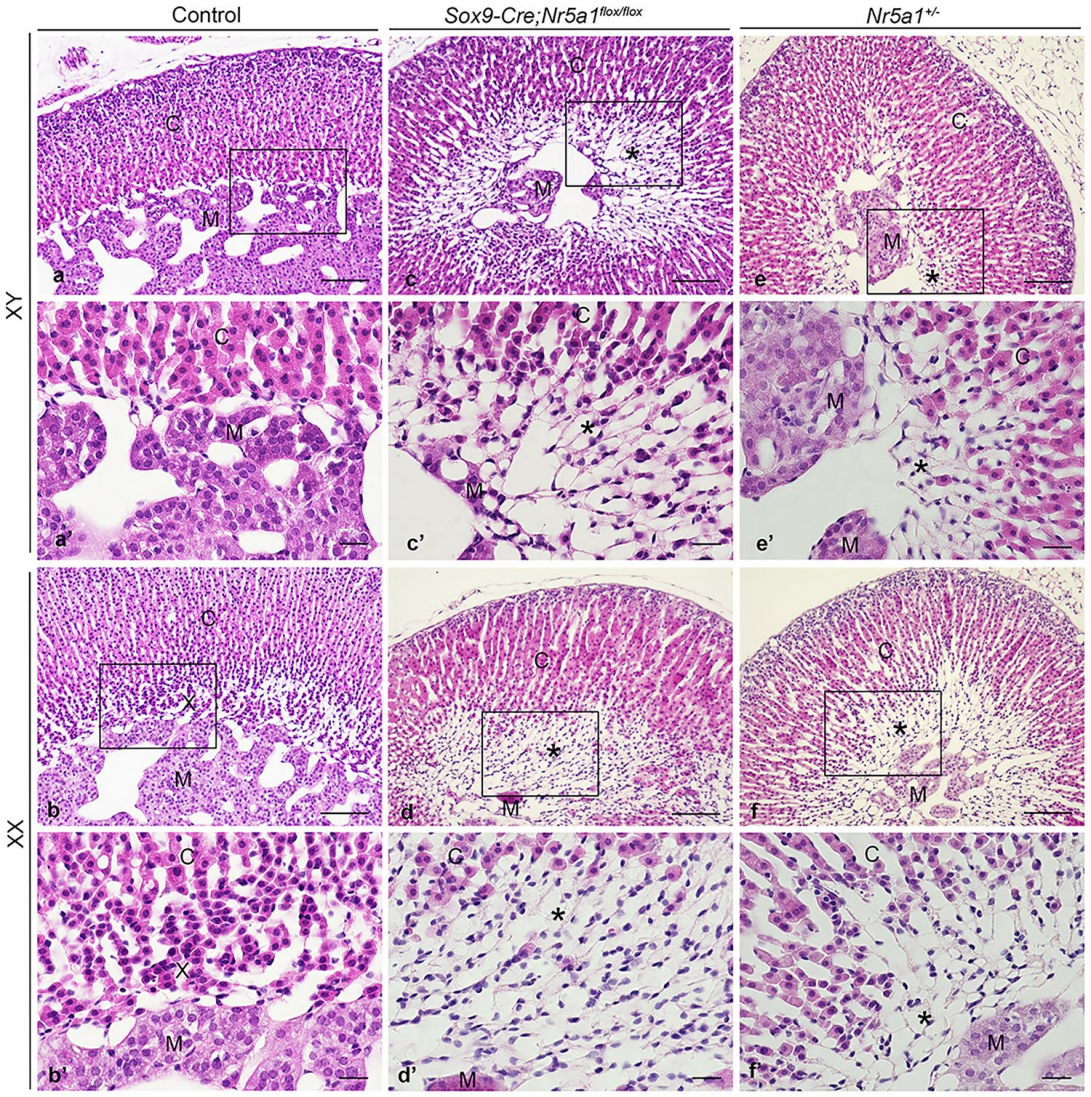
Histology in adult adrenals. Representative images of hematoxylin and eosin staining performed on adrenal sections from control (**a**, **b**), *Sox9-Cre;Nr5a1*^*flox/flox*^ (**c**, **d**), and *Nr5a1*^+*/−*^ (**e**, **f**) mice of males (XY) and females (XX) at 8 weeks of age. **a**′–**f**′ are higher magnification views of the regions indicated by the box in **a**–**f**, respectively. * indicates the area, in which cells are devoid of eosin and vacuolated. C, cortex; M, medulla; X, X-zone. Scale bars, 100 µm in **a**–**f**; 20 µm in **a**′–**f**′.

Hematoxylin and eosin (HE) staining of adrenal sections was performed for histological analysis. In all three genotypes, the ZG and ZF were distinguished in the outer and inner cortices, respectively, and the thickness of the cortex was similar (Fig. 8). The 20αHSD-positive X-zone cells, which were observed in control female adrenals, were eosinophilic without vacuolation (Fig. 8b, b′), while the 20αHSD-negative area at the innermost cortex in *Sox9-Cre;Nr5a1*^*flox/flox*^ and *Nr5a1*^+*/−*^ adrenals was filled with eosin-negative vacuolated cells (Fig. 8c–f, c′–f′, indicated by *).

## Discussion

In this study, we first confirmed that SOX9 is expressed by adrenocortical cells at E10.5–E11.5, but is extinguished no later than E12.5 (Fig. 1;^16^), and demonstrated that a drastic reduction in adrenocortical cells occurs at E11.5–E12.5 due to *Sox9-Cre*-directed *Nr5a1* cKO. Therefore, the abrogated adrenal development in *Sox9-Cre;Nr5a1*^*flox/flox*^ mice reflects the loss of fetal adrenocortical cells by *Nr5a1* disruption.

In *Nr5a1*^*−/−*^ mice, both AP and GP regressed at E11.5–E12 due to apoptosis^7^. Similarly, increased apoptosis was detected in the *Sox9-Cre;Nr5a1*^*flox/flox*^ AP than in controls at E12.5. The results support the idea that Nr5a1 plays an important role in adrenal cell survival by regulating apoptosis^10,17^. However, in *Nr5a1*^+*/−*^ mice, increased apoptosis was not detected at E11.5–E12.5. The results did not differ from those of a previous study, in which the number of TUNEL-positive cells did not differ between the control and *Nr5a1*^+*/−*^ AP at E11.0–E12.0, since fewer number of cells in the AGP were dedicated to adrenocortical progenitors at E10.0^8^. E12.5 corresponds to the time at which AP expansion and events for adrenal organogenesis, including medulla formation and encapsulation, begin. *Nr5a1*^*−/−*^ mice show complete agenesis of the adrenals and die soon after birth due to adrenal insufficiency^7^, whereas *Sox9-Cre;Nr5a1*^*flox/flox*^ mice develop hypoplastic adrenals in adulthood. Therefore, *Sox9-Cre;Nr5a1*^*flox/flox*^ mice are practically useful for investigating the importance of Nr5a1 specifically at the critical stage of adrenal development.

The cell proliferation rate did not differ between *Sox9-Cre;Nr5a1*^*flox/flox*^ mice and the controls at E13.5 or E15.5. Fetal-type adrenocortical cells begin to regress around E13.5, and are centripetally displaced by definitive-type adrenocortical cells; they emerge at the boundary between the capsule and the cortex, and proliferate in the outer cortex during embryonic development^18^. Since BrdU-positive proliferating cells were localized in the outer cortex, cell proliferation of *Sox9-Cre;Nr5a1*^*flox/flox*^ adrenals should occur mainly in definitive adrenocortical cells at E15.5. Nr5a1 has been thought to have a role in cell proliferation in the definitive cortex^9^. It has been reported that in *Nr5a1*^+*/−*^ mice, the proliferation rate of adrenocortical cells did not differ from that in control embryos at E12.5, but showed a significant increase compared to controls after E13.5, suggesting that the signals of fetal adrenocortical cell loss activated the factors involved in cell proliferation and indirectly induced the proliferation of definitive adrenocortical cells by an unknown mechanism^8^.

The adrenal size in *Sox9-Cre;Nr5a1*^*flox/flox*^ mice did not match that of the controls during embryonic development through adulthood. Despite the similar proportions of proliferating adrenocortical cells between *Sox9-Cre;Nr5a1*^*flox/flox*^ and control mice at E13.5-E15.5, the drastic loss of fetal adrenocortical cells in *Sox9-Cre;Nr5a1*^*flox/flox*^ mice by E12.5 might cause displacement of definitive-type adrenocortical cells or affect the generation of definitive cortical cells. Capsular cells, which originate from fetal cortical cells, have been reported to serve as stem cells of definitive cortical cells^18–21^. The smaller size of the adrenal gland in *Sox9-Cre;Nr5a1*^*flox/flox*^ mice may be related to the delayed capsule formation.

The results of IF for TH showed normal arrival of TH-positive cells in the AP area at the proper time in *Sox9-Cre;Nr5a1*^*flox/flox*^ mice, indicating that the migration of neural crest cells into the AP is not be dependent on *Nr5a1*^22,23^. However, only a small number of TH-positive cells invaded the cortex of *Sox9-Cre;Nr5a1*^*flox/flox*^ adrenals. The AP size and the trend of TH-positive cells are similar between *Sox9-Cre;Nr5a1*^*flox/flox*^ and *Nr5a1*^+*/−*^ mice. Therefore, as seen in *Nr5a1*^+*/−*^ mice, incomplete penetration of TH-positive cells into the cortex may be associated with hypoplastic AP, suggesting that an appropriate size of the AP is needed for the invasion of TH-positive cells into the cortex, and that signals from a sufficient number of adrenocortical cells may be required for the invasion of TH-positive cells.

The phenotypes of *Sox9-Cre;Nr5a1*^*flox/flox*^ adrenals were similar to those of *Nr5a1*^+*/−*^ mice during embryonic development, but differed in adulthood.

*Sox9-Cre;Nr5a1*^*flox/flox*^ and *Nr5a1*^+*/−*^ adrenals were both hypoplastic, with similar sizes at 8 weeks of age. Medulla organization in *Sox9-Cre;Nr5a1*^*flox/flox*^ mice was impaired, but the severity varied across individual adrenals. The variable phenotypes of *Sox9-Cre;Nr5a1*^*flox/flox*^ adrenal glands might reflect subtle differences in the extent of *Nr5a1* disruption, leading to different levels of adrenocortical cell loss in individuals. In contrast, *Nr5a1*^+*/−*^ adrenal glands maintained a relatively normal medulla organization.

The presence or absence, morphology, and thickness of the X-zone differ depending on sex, strain, and age^15^. Although the X-zone shows sexually dimorphic regression under normal conditions, 20αHSD-positive X-zone cells were present in *Sox9-Cre;Nr5a1*^*flox/flox*^ mice of both sexes. X-zone regression is dependent on androgens, and androgen signaling via the androgen receptor is essential for X-zone regression in both male and female adrenal glands^24,25^. Persistence of the X-zone in *Sox9-Cre;Nr5a1*^*flox/flox*^ males is probably due to low testosterone levels since the gonads in *Sox9-Cre;Nr5a1*^*flox/flox*^ males were hypoplastic and exhibited aberrant ovotestis structures. Circulating testosterone levels in *Sox9-Cre;Nr5a1*^*flox/flox*^ mice were significantly lower than those in controls (Supplementary Fig. 2). In contrast, *Nr5a1*^+*/−*^ male mice that developed normal testes lacked the X zone. These results confirmed that testicular testosterone is at least a factor involved in X-zone regression in males.

Vacuolated cells were detected in the innermost cortex of *Sox9-Cre;Nr5a1*^*flox/flox*^ and *Nr5a1*^+*/−*^ adrenals. In terms of morphology, vacuolated and non-vacuolated cell types have been observed in the X zone, and the presence or absence of the vacuolated X zone differs depending on various factors, including the mouse strain, sex, age, and physiological conditions, such as virgin or parous^26,27^. In a study by Tanaka and Matsuzawa^26^, vacuolated X-zone cells were observed in DDD virgin females at ages 35 and 70 days, but not in age-matched mice of C57BL/6 J, which was the mouse strain used in this study. The vacuolated cells detected in *Sox9-Cre;Nr5a1*^*flox/flox*^ and *Nr5a1*^+*/−*^ adrenal glands morphologically resembled the vacuolated X-zone cells. However, they lacked the expression of both 3βHSD and 20αHSD, and seemed different from the so-called X-zone cells, which are positive for both of the above. Vacuolation in the X-zone is thought to be related to cellular degeneration^27^. In BalB/c mice, X-zone regression in pregnant females is accompanied by the loss of 20α-HSD activity and development of lipid vacuoles^28^. Therefore, the vacuolated cells could be in the process of X-zone degeneration, although X-zone cells in C57BL/6 J mice have been reported to be degenerate without vacuolation^26^. To determine whether the vacuolated area was associated with cellular degeneration, we performed IF for CC3; however, no difference in the location or frequency of apoptotic cells was detected among the genotypes (Supplementary Fig. 3). Furthermore, the vacuolated cell area was detected regardless of males or females in both *Sox9-Cre;Nr5a1*^*flox/flox*^ and *Nr5a1*^+*/−*^ adrenals, indicating that the area is independent of sex hormones; however, it seemed to be associated with early loss of *Nr5a1*.

Altogether, our results revealed that the loss of adrenocortical cells by *Sox9-Cre-*directed *Nr5a1* disruption affects adrenal development. *Sox9* is known to be expressed in progenitor populations during the embryonic development of chondrocytes; Sertoli cells; upper digestive tract organs (e.g., liver, pancreas, and duodenum); and organs such as the heart, lung and trachea, central nervous system (CNS), skin, hair follicles, retina, and inner ear, thus, playing essential roles in morphogenesis^14^. In this study, SOX9 expression was observed in adrenocortical cells during a limited critical period of adrenal development, which suggested the involvement of Sox9 in adrenal development. The different phenotypes of *Sox9-Cre;Nr5a1*^*flox/flox*^ and *Nr5a1*^+*/−*^ adrenal glands may reflect the absence or presence of NR5A1/SOX9-positive cells at the beginning of adrenal organogenesis. Further studies would be required to determine the possible interactions between Nr5a1 and Sox9 during adrenal development.

## Methods

All methods were performed in accordance with the ARRIVE guidelines.

### Animals

All procedures were conducted in accordance with the National Institutes of Health Guide for the Care and Use of Research Animals and were approved by the Ethical Committee for Animal Use of the Aichi Gakuin University.

*Sox9-Cre;Nr5a1*^*flox/flox*^ mice had been described previously and were bred on a C57BL/6 strain background^12^. All mice had free access to water and standard rodent chow and were housed under 12-h light/12-h dark cycles. The mice were genotyped by PCR using specific primer sets and sequences. We defined the day when a vaginal plug was identified as E0.5. *Nr5a1*^*flox/flox*^, *Sox9-Cre;Nr5a1*^*flox/*+^, or *Nr5a1*^*flox/*+^ littermates were used as controls.

### Histology, immunohistochemistry (IHC), and immunofluorescence (IF)

Pregnant mice were anesthetized in a chamber containing 5% isoflurane oxygen and euthanized by cervical dislocation. Embryos obtained at E11.5 to E18.5 stages were fixed overnight in 4% paraformaldehyde (PFA) at 4 °C. Postnatal animals were anesthetized and perfusion-fixed with 4% PFA, their adrenals were dissected, and post-fixed in 4% PFA at 4 °C overnight. Both embryos and postnatal adrenals were embedded in paraffin, and sectioned at 6-μm thickness. The tissue sections were de-paraffinized and rehydrated using a series of xylene/alcohol solutions.

For histological analysis, the sections were stained with hematoxylin and eosin (HE) following standard procedures.

For IHC and IF, antigen retrieval was performed via microwave boiling for 5 min in 0.01 M citrate or Histofine antigen retrieval pH9.0 (415211; Nichirei Biosciences). Endogenous peroxidase activity was quenched by immersing sections in 3% (v/v) hydrogen peroxide in phosphate-buffered saline (PBS) for 15 min. After three washes with PBS, the sections were incubated with 5% (w/v) Block Ace (Dainippon Pharmaceutical) at 20 °C for 30 min, followed by that with primary antibodies at appropriate dilutions overnight at 4 °C. The primary antibodies were rabbit anti-NR5A1^12^, rabbit anti-SOX9 (ab185230; Abcam), rabbit anti-3βHSD (KO607; Trans Genic), rabbit anti-20αHSD/AKR1C1 (OAGA00409; Aviva Systems Biology), rabbit anti-Tyrosine Hydroxylase (AB152; Millipore), rabbit anti-Cleaved Caspase-3 (9661; Cell Signaling), goat anti-GATA4 antibody (sc1237; Santa Cruz Biotechnology), mouse anti- NR2F2/COUP-TFII antibody (PP-H7147; Perseus Proteomics), and mouse anti-BrdU antibody (B8434; Sigma-Aldrich). Sections for IHC were washed with PBS containing 0.1% (v/v) Triton X, followed by incubation with either N-Histofine Simple Stain Mouse MAX PO (R) (Nichirei Biosciences). The peroxidase reaction was visualized using a Vector NovaRED (Vector Laboratories). The sections were dehydrated and mounted using Mount-Quick (Hokkaido Sangyo). Double-label IF was performed by co-incubating polyclonal rabbit antibodies with monoclonal mouse or polyclonal goat primary antibodies. Sections were then incubated with donkey anti-mouse, anti-goat, and anti-rabbit secondary antibodies conjugated with Alexa 488 and Alexa 555 for 2 h, and then washed with PBS. Nuclei were counterstained with 4,6-diamidino-2-phenylindole (DAPI; Sigma-Aldrich) and mounted using Fluoromount (Diagnostic BioSystems). Triple-label IF was performed using the Opal 4-Color Automation IHC Kit (Akoya Biosciences) according to the manufacturer’s instructions.

Adrenal samples from at least three animals in each genotype group at each time point were processed in parallel to reproducibly compare the immunostaining results on at least two separate occasions. Negative-control tissue sections were incubated without primary antibodies. Digital images were acquired using a Keyence All-in-One Fluorescence Microscope (BZ-X710) and transferred to Photoshop CS (Adobe Systems) to generate the figures. Adjustments for the contrast and brightness, and image cropping for presentation were performed to the same degree for all images. For quantitative evaluation of the immunostaining data, at least three adrenals from three mice per genotype were used for cell counting.

The number of NR5A1-positive and BrdU-positive cells per section was measured using the ImageJ Fiji program. The number of digitally counted NR5A1-positive and BrdU-positive cells was confirmed by visual assessment to ensure appropriate parameter settings.

Adrenal sizes were measured using HE-stained sections. Digital images were acquired using a Keyence All-in-One Fluorescence Microscope (BZ-X710), and adrenal sizes were measured using the ImageJ Fiji program.

### Bromodeoxyuridine (BrdU) incorporation

For proliferation analysis, 50 mg/kg body weight BrdU (ab142567; Abcam) was injected intraperitoneally into pregnant mice. Two hours after injection, the pregnant females were sacrificed, and the embryos were dissected, fixed overnight in 4% PFA at 4 °C, and embedded in paraffin using standard procedures.

Data are expressed as percentages of the number of double-positive cells for NR5A1 and BrdU relative to the total number of NR5A1-positive cells. In the AP, three sections were analyzed per animal (n = 3).

### Blood testosterone level

Mice at 8 weeks of age were anesthetized in a chamber containing 5% isoflurane oxygen and euthanized via cervical dislocation. Cardiac blood samples were collected. Serum testosterone concentrations were measured using a Testosterone rat/mouse ELISA Kit (DEV9911; Demeditec), according to the manufacturer's protocol.

### Statistical analysis

Statistical differences were assessed using Student’s t-test. Differences were considered significant at P < 0.05. All data are reported as means ± SEM.

### Supplementary Information

Supplementary Figures.

## Data Availability

The datasets used and analyzed during the current study are available from the corresponding author upon reasonable request.
