# Supplementary material for: Conditional disruption of Nr5a1 directed by Sox9-Cre impairs adrenal development
Source: Sci Rep. 2024 May 29;14:12297. doi: 10.1038/s41598-024-63264-9 (PMC11137078; doi:10.1038/s41598-024-63264-9)
Supplement: Supplementary file 1 — Supplementary Figures. [file 41598_2024_63264_MOESM1_ESM.pdf]

Conditional disruption of *Nr5a1* directed by *Sox9-Cre* impairs adrenal development

Ayako Tagami, Yayoi Ikeda*, Kyoko Ishizuka, and Mamiko Maekawa

Department of Anatomy, Aichi Gakuin University School of Dentistry, Nagoya, Japan

***Corresponding author:**
Yayoi Ikeda, DDS, PhD
Department of Anatomy, School of Dentistry, Aichi Gakuin University
1-100 Kusumoto-cho, Chikusa-ku, Nagoya, Aichi 464-8650, Japan
Tel: +81-52-757-6756
Fax: +81-52-757-6755
E-mail: yayoi@dpc.agu.ac.jp

Supplementary Figure 1

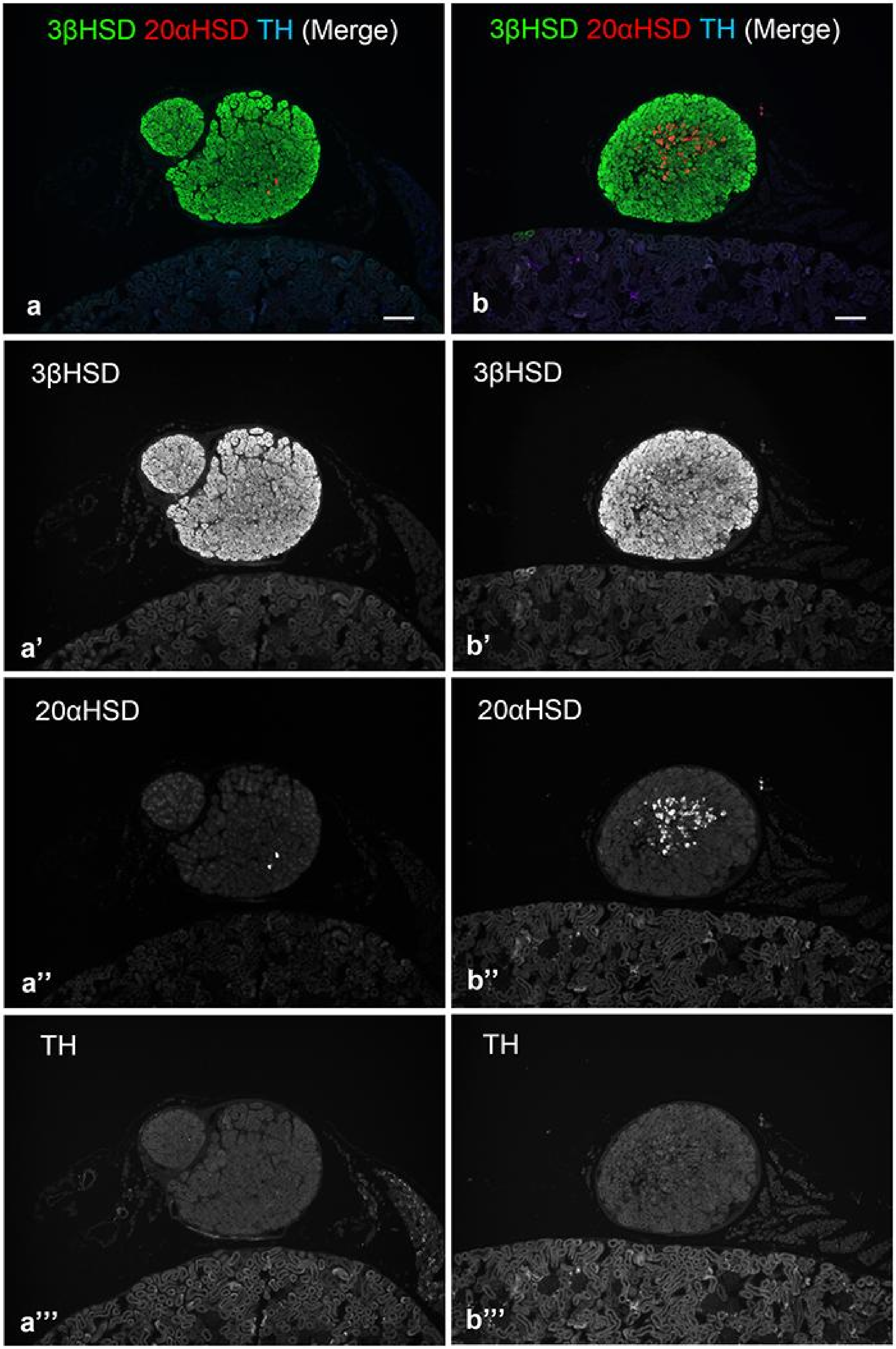

Supplementary Figure 1. *Sox9-Cre;Nr5a1*$^{flox/flox}$ adrenals without medulla. (**a**, **b**) Merged images of triple-label immunofluorescence for the steroidogenesis marker 3βHSD (green, **a'**, **b'**), the X-zone marker 20αHSD (red, **a''**, **b''**), and the medulla marker TH (blue, **a'''**, **b'''**) in sections from two *Sox9-Cre;Nr5a1*$^{flox/flox}$ adrenals at 8 weeks of age. No immunoreactivity for TH was detectedn in either one of the two *Sox9-Cre;Nr5a1*$^{flox/flox}$ adrenals. Scale bars, 100 µm.

Supplementary Figure 2

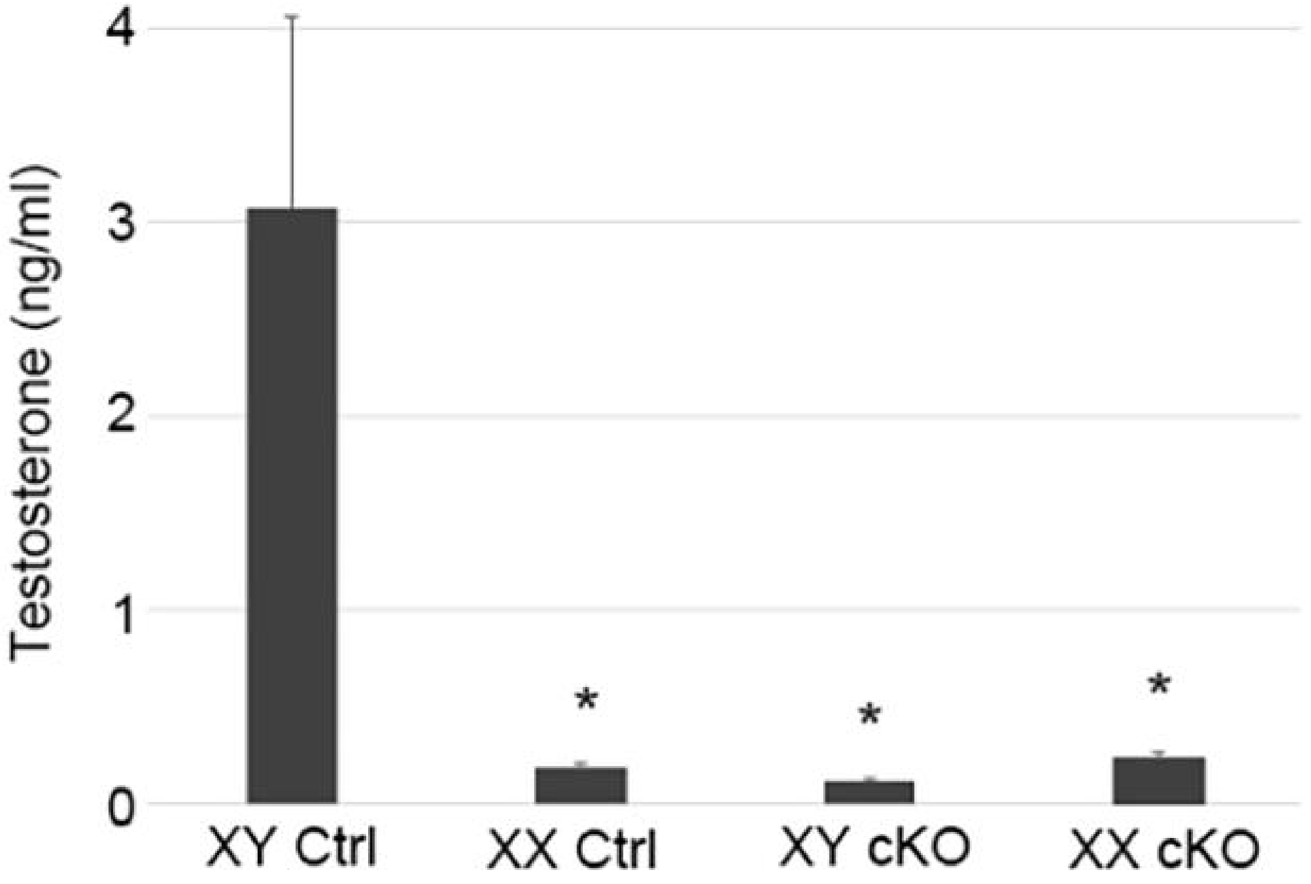

Supplementary Figure 2. Blood testosterone level. Testosterone levels in XY *Sox9-Cre;Nr5a1*$^{flox/flox}$ (XY cKO) mice were significantly lower compared to those in XY control (XY Ctrl) mice, and were similar to those in XX Ctrl and XX cKO at 8 weeks of age. Data are shown as means ± SEM (n = 3). *$P < 0.05$ vs. XY Ctrl.

Supplementary Figure 3

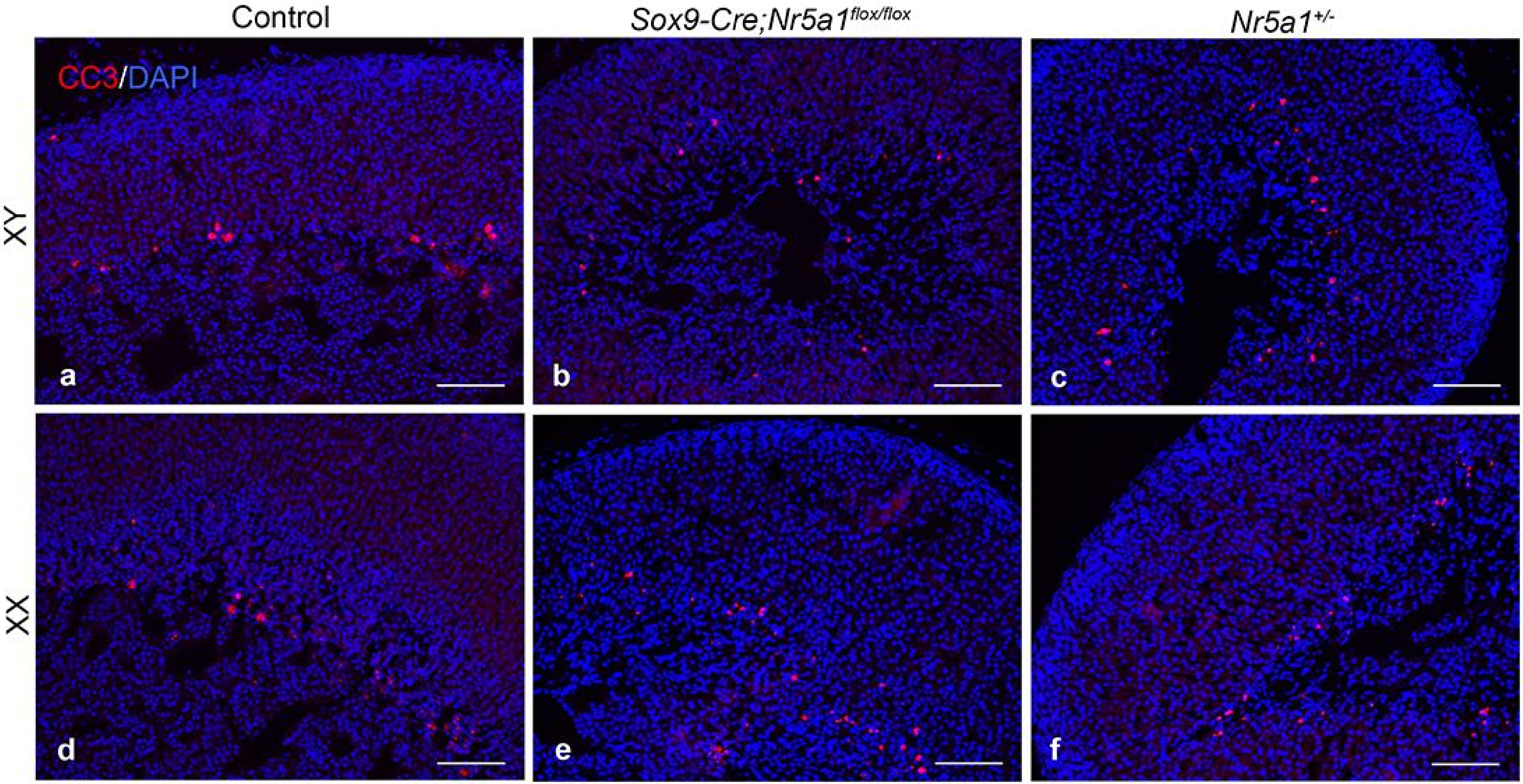

Supplementary Figure 3. Apoptosis in adult adrenals. Immunofluorescence for cleaved caspase-3 (CC3, red) in adrenal sections from control, *Sox9-Cre;Nr5a1*$^{flox/flox}$, and *Nr5a1*$^{+/-}$ mice at 8 weeks of age. Nuclei are stained blue with DAPI. **a**-**f** are adjacent serial sections of **a**-**f** in Figure 8, respectively. Scale bars, 100 µm.
